# Sea urchin intestinal bacterial communities depend on seaweed diet and contain nitrogen-fixing symbionts

**DOI:** 10.1093/femsec/fiaf006

**Published:** 2025-01-14

**Authors:** Mia M Bengtsson, Marita Helgesen, Haitao Wang, Stein Fredriksen, Kjell Magnus Norderhaug

**Affiliations:** Institute of Microbiology, University of Greifswald, Felix-Hausdorff-Straße 8, 17489 Greifswald, Germany; Institute of Marine Biotechnology, Walther-Rathenau-Str. 49A, 17489 Greifswald, Germany; Institute of Marine Research IMR, Nye Flødevigveien 20, 4817 His, Norway; Department of Biosciences, University of Oslo, Blindernveien 31, 0371 Oslo, Norway; Institute of Microbiology, University of Greifswald, Felix-Hausdorff-Straße 8, 17489 Greifswald, Germany; Institute of Marine Research IMR, Nye Flødevigveien 20, 4817 His, Norway; Department of Biosciences, University of Oslo, Blindernveien 31, 0371 Oslo, Norway; Institute of Marine Research IMR, Nye Flødevigveien 20, 4817 His, Norway

**Keywords:** grazing, gut microbiome, kelp forest, macroalgae, nitrogen fixation, sea urchin

## Abstract

Kelp deforestation by sea urchin grazing is a widespread phenomenon globally, with vast consequences for coastal ecosystems. The ability of sea urchins to survive on a kelp diet of poor nutritional quality is not well understood and bacterial communities in the sea urchin intestine may play an important role in digestion. A no-choice feeding experiment was conducted with the sea urchin *Strongylocentrotus droebachiensis*, offering three different seaweeds as diet, including the kelp *Saccharina latissima*. Starved sea urchins served as experimental control. Amplicons of the 16S rRNA gene were analyzed from fecal pellets. One dominant symbiont (*Psychromonas marina*) accounted for 44% of all sequence reads and was especially abundant in the sea urchins fed seaweed diets. The starved and field-captured sea urchins consistently displayed higher diversity than the seaweed-fed sea urchins. Cloning and sequencing of the *nifH* gene revealed diverse nitrogen fixers. We demonstrate that the sea urchin intestinal microbiome is dynamic, with bacterial communities that are plastic, depending on diet and have the capacity for nitrogen fixation. This reflects the dietary flexibility of these sea urchins, and their intestinal microbiota could be a key component in understanding catastrophic kelp forest grazing events.

## Teaser

Gut bacterial communities vary with the seaweed fed to sea urchins.

## Introduction

Sea urchins are keystone species in coastal ecosystems and a prominent grazer transforming highly productive kelp forests into unproductive marine deserts (Filbee-Dexter and Scheibling 2014). In the past century, kelp forests in many parts of the world have been turned into so-called “barren grounds” due to excessive grazing by sea urchins (Bernstein et al. 1981, Scheibling et al. 1999, Norderhaug and Christie 2009). For example, grazing by the sea urchin *Strongylocentrotus droebachiensis* led to the loss of 2000 km^2^ of *Laminaria hyperborea* and *Saccharina latissima* kelp forest in northern Norway from the 1970s until today (Norderhaug et al. 2021). The barren ecosystem can persist for decades, due to constant grazing by sea urchins, which prevents the regrowth of kelp. When the kelp forest ecosystem is transformed into barren ground, a significant loss in biodiversity and productivity will follow, and numerous important ecosystem services will be lost (Eger et al. 2023). One ecosystem service that has received increasing attention during the last decade is the role of kelp forests in climate mitigation by their high efficiency in capturing CO_2_ and what is referred to as “blue carbon” (Krause-Jensen et al. 2018). Large quantities of fragmented and dead kelp detritus are transported to deeper water, buried in sediments, and thereby removed from the carbon cycle. Sea urchins have a significant role in this carbon sink function. They can consume a large part of the drift kelp on shallow water (Filbee-Dexter et al. 2019), and by transforming drift kelp into sea urchin pellets, increase the dispersal potential and extend carbon export into deep water (Wernberg and Filbee-Dexter 2018).

Sea urchins are omnivores as they consume a broad diversity of organisms from several trophic levels, yet, their primary food source is seaweed like kelp (Himmelman and Steele 1971). The ability to live on fresh kelp tissue is rare among animals, and few organisms other than sea urchins can live directly from fresh kelp (Mann 1977). Kelp is difficult to digest because of complex carbohydrates such as alginate that are not readily decomposed by animal enzymes (Lasker and Giese 1954), and the lack of protein. This results in a carbon to nitrogen (C:N) ratio exceeding what is found in most marine organisms, which means that kelp has poor nutritional quality (Sterner and Hessen 1994, Norderhaug et al. 2003). In order to survive on such an unpalatable diet, sea urchins require adaptations to digest complex carbohydrates and an ability to compensate for the lack of proteins. Other organisms that live on carbon-rich materials can give some indications to how sea urchins can survive on a kelp-dominated diet. For example, shipworms have established symbiotic relationships with bacteria in their guts that makes it possible to live on wooden materials (Lechene et al. 2007, Betcher et al. 2012). Some of these bacteria decompose complex carbohydrates and others fix nitrogen into biologically available ammonia (NH_3_), thus compensating for the lack of protein in the food (Lechene et al. 2007). Bacteria with similar functions are likely beneficial for sea urchins as well. In fact, nitrogen fixation has been detected in association with some sea urchins, including *S. droebachiensis* (Guerinot and Patriquin 1981a) and a nitrogen-fixing *Vibrio* sp. strain was isolated in culture from the same species (Guerinot and Patriquin 1981b). In addition, bacteria with the ability to degrade the important kelp cell wall polysaccharide alginate have been isolated from sea urchin intestines (Sawabe et al. 1995).

Despite these early findings, the possible role of the sea urchin microbiome in catastrophic kelp forest grazing events has received relatively little attention. One recent study investigated the intestinal microbiome of *Strongylocentrotus purpuratus* and found that microbiota was highly variable between gut tissue and gut digesta (Hakim et al. 2019), indicating a spatial separation in microbial niches in the gut. The intestinal microbiota of *S. purpuratus* was also found to be distinct from another co-occurring sea urchin species, and varied depending on whether it was sampled from kelp beds or from barren grounds (Miller et al. 2021). Similarly, the intestinal microbiota of another sea urchin, *Mesocentrotus nudus*, differed depending on barren ground severity (Park et al. 2023). *Mesocentrotus nudus, S. purpuratus*, and *S. droebachiensis* all cause catastrophic grazing of kelp forests, and the latter is a quantitatively relevant grazer of kelp in the entire northern hemisphere (Filbee-Dexter and Scheibling 2014).

In this study, we set out to fill critical knowledge gaps in the understanding of sea urchin grazing by investigating the bacterial component of intestinal microbiota of *S. droebachiensis* (green sea urchin) during grazing and digestion of seaweeds. We experimentally exposed sea urchins to different seaweed diets and hypothesized that: (1) different seaweed as a food source lead to different intestinal microbiota composition during food degradation and that (2) bacterial N_2_-fixing symbionts are present in sea urchin intestines, which may help the host to utilize seaweed biomass with a high C:N ratio. We used 16S rRNA gene amplicon sequencing to study differences in bacterial community composition and diversity between different food sources. Cloning and sequencing of the *nifH* gene was used to detect and identify potential nitrogen-fixing members of the *S. droebachiensis* intestinal microbiota.

## Materials and methods

### Sea urchin collection and experimental design

**Figure 1.**
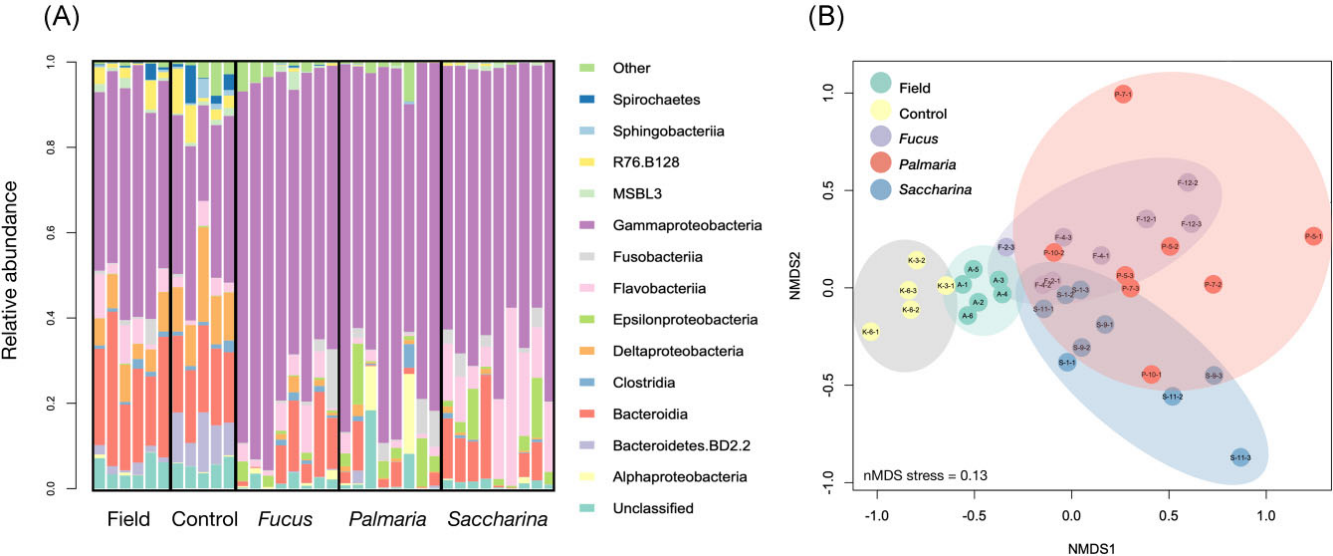
Sea urchin intestinal bacterial community composition depended on food availability. (A) Gammaproteobacteria dominated most samples, especially in the treatments where urchins were fed seaweed (seaweed-fed: *Fucus, Palmaria*, and *Saccharina*). (B) The nMDS plot shows a separation between starved (control) and field-collected (natural diet control) versus seaweed-fed treatments. The shaded ellipses have been added to aid in interpretation and do not represent confidence intervals.

Sea urchins (*S. droebachiensis* O.F. Müller) ~40–60 mm in diameter were collected next to Hallangstangen (59°40′58.8″N, 10°36′49.0″E; Fig. 1), 4 km north of the main harbor in Drøbak (Norway) on 4 January 2017. As sea urchins are mostly found on hard substrata where they can be firmly attached to the surface, a triangular dredge was used to collect the sea urchins from a depth of 6–15 m.

For the experiment, sea urchins were randomly distributed among 12 tanks (L × W × H: 35 cm × 20 cm × 25 cm = 17.5 l) with five individuals in each tank (5 sea urchins × 12 tanks = 60 sea urchins). The set-up was designed as a flow-through system, where each tank had its own filter transporting new seawater from a large container, that was continuously refilled from an inlet next to the Biological station in Drøbak. This design was selected to prevent mixing of water among tanks, and to provide natural water conditions for the sea urchins. The rate of water flow was between 33 and 67 l/h. The sea urchins were kept in the tanks and starved for 10 days prior to implementing the experimental treatments, to minimize the influence of previous feeding and to let them acclimatize to the new conditions. Water temperature (ranging between 3.5°C and 5.4°C) and salinity (26.2–30.3 ppt) were monitored on a regular basis to assure good conditions for the sea urchins. The lights were turned off during the experiment (except when handling), to reduce the impact of undesirable growth by algae in the tanks.

### Experimental treatments

A no-choice feeding experiment was conducted to determine the influence of diet on the bacterial intestinal microbiota. The diets consisted of three seaweed species: *S. latissima* (Linnaeus) C.E. Lane, C. Mayes, Druehl and G.W. Saunders, *Fucus serratus* (Linnaeus), and *Palmaria palmata* (Linnaeus) F. Weber and D. Mohr. These algae were selected as they were present in the area, where the sea urchins were collected. *S. latissima* (treatment “*Saccharina*”) is the dominating kelp on sheltered coasts, and the other two algae (treatments “*Fucus*” and “*Palmaria*”) were selected as they generally have different nutrient and chemical profiles compared to kelp, and can function as an alternative food source when kelp is absent. Control tanks (treatment “Control”) with sea urchins, which were not fed (starved) were also set up to evaluate if the experimental conditions impacted the results. In addition, samples were taken directly from the collected sea urchins at the field site before initiating the experiment, to represent a natural state (treatment referred to as “Field”). The seaweed used as feed was sampled prior to the experiment to estimate the carbon and nitrogen content, as a proxy for nutritional quality, using an elemental analyzer (Thermo Finnigan).

### Intestinal microbiota sample collection

After being exposed to the experimental treatments for 10 days, sea urchin intestinal microbiota were assessed by collection of fecal pellets directly from the large intestine of dissected sea urchins. This method was preferred over collecting fecal pellets in the experimental tanks, to avoid contamination and degradation of fecal material by the surrounding water, and to assign the sampled material to distinct individuals. Intestinal tissue was also collected for comparative purposes. Fecal pellet samples were taken from three sea urchins from each experimental tank (3 sea urchins × 12 tanks = 36 samples), in addition, samples were taken from sea urchins before the experiment (six individuals, “Field” treatment). The sea urchins were dissected and fecal pellets from the large intestine were placed into separate cryovials and then frozen directly with liquid nitrogen to prevent genomic DNA from degrading. Between each dissection, the equipment was sterilized with decanox, sterilized water, and ethanol (70%). The samples were stored at −80°C until DNA extraction. Fecal pellets could not be found in all sea urchins, especially the starved (Control) individuals, thus the sample size in all groups was not identical.

### DNA extraction and analysis

Extraction of genomic DNA was performed with the commercial DNeasyPowerSoil® Kit (Qiagen, formerly MoBio Laboratories). The fecal pellet samples were thawed and kept on ice and samples were handled as fast as possible, since pilot experiments had shown that DNA degradation was a critical issue. The DNA extraction was carried out according to the protocol provided by the manufacturer, with some minor changes: Solution C1 was added before the sample, the amount of fecal pellet samples added was between 0.02 and 0.08 g, and half the amount of solution C6 was used to elute the extracted DNA in the final step. The concentration of DNA extracted from each sample was measured with a Qubit 3.0 fluorometer.

Polymerase chain reaction (PCR) and Illumina MiSeq V3 sequencing were carried out by LGC Genomics in Germany. The primers used were: forward: 515F-mod (5′-GTGYCAGCMGCCGCGGTAA-3′) and reverse: 806R-mod (5′-GGACTACNVGGGTWTCTAAT-3′) (Walters et al. 2016), targeting the V4 region of the prokaryotic 16S rRNA gene, and linked to custom barcode and adaptor constructs. The sequence data has been uploaded to the European Nucleotide Archive under the accession number PRJEB54963.

### 16S rRNA gene amplicon sequence processing

The DADA2 R package (Callahan et al. 2016; version 1.4.0) was used to quality-filter and assign amplicon sequence variants (ASVs) from the 16S rRNA amplicon sequencing data. Briefly, sequence reads (primer sites clipped away) were truncated to 180 bp, filtered (maxN = 0, maxEE = 2, and truncQ = 2) and dereplicated. Error rates were estimated using the maximum possible error estimate from the data as an initial guess. Sequences were inferred, and paired forward and reverse reads were merged allowing a minimum overlap of 12 bp and 0 mismatches. Only merged sequences with a length between 251 and 254 bp were considered. Chimeric sequences were removed using the removeBimeraDenovo function. Taxonomic classification of the ASVs generated by DADA2 was carried out using a lowest common ancestor approach in CREST (Lanzen et al. 2012), using the silvamod v128 database, based on SILVA taxonomy (Pruesse et al. 2007). ASVs classified as eukaryotic (Domain/level1 = “Eukaryota”), which comprised algal plastid ASVs and mitochondrial ASVs, were removed prior to all downstream analyses except for the differential abundance analysis.

### Statistical analysis of 16S rRNA gene amplicon sequencing data

All statistical analyses were carried out using R (version 3.4.1) (R Development Core Team 2023). Prior to analysis, four samples (one *Control*-, one *Fucus*-, and one *Palmaria* treatment) were removed due to low sequencing depth (<10 000 reads after removal of eukaryotic ASVs). ASV richness (S) was calculated using sequence data rarefied to the lowest sequencing depth for the remaining samples (17 650) using functions *rarefy* and *diversity* from the vegan package (Oksanen et al. 2016). To test for differences in richness and evenness between treatments, analysis of variance (ANOVA, *aov* function) followed by a Tukey *post hoc* test (Tukey HSD function) was carried out. Richness data was log transformed to achieve normality.

To analyze the bacterial community composition, multivariate statistics as implemented in the vegan R package (Oksanen et al. 2016) were performed. ASV data was Hellinger-transformed with the *decostand* function. The transformed values were used as basis for non-metric multidimensional scaling (nMDS, *metaMDS* function) plot and permutation analyses of variance (PERMANOVA, *adonis2* function). As there is a risk of sea urchins within the same tank having similar community composition, and that there might be an interaction between treatment and tank variables, an interaction term (treatment × tank) was added to the formula for the PERMANOVA. A pairwise PERMANOVA (*pairwise.adonis2* function, pairwiseAdonis package) with a following *P*-value adjustment (*p.adjust* function, Bonferroni method) was used to test for differences in community composition between individual treatments.

A differential abundance analysis was performed using the R package DESeq2 (Love et al. 2014) to identify bacterial ASVs that had a significant differential relative abundance in the different treatments. This analysis was performed on the entire dataset, without prior removal of eukaryotic ASVs. Pairwise comparisons of each of the seaweed treatments against the Control treatment (starved sea urchins), as well as of the Field treatment (field captured) against the Control, were made. Only ASVs with an adjusted *P*-value of < .01 were considered significantly differentially abundant.

### Analysis of N_2_-fixation potential via *nifH* gene sequencing

We amplified a fragment of the *nifH* gene from a subset of the samples using the IGK3/DVV PCR primers (Ando et al. 2005) and subsequently cloned these fragments into competent *Escherichia coli* cells using TOPO TA cloning. Clones were sequenced using Sanger sequencing technology and the sequences were submitted to GenBank under accession numbers OP380433–OP380446. The obtained DNA sequences were translated to protein sequences, which were used as query sequences for searching against the NCBI protein database. Reference sequences were chosen from highly similar sequences to each deduced protein sequence. A maximum-likelihood phylogenetic tree was built with the deduced and reference sequences using MEGA7 (Kumar et al. 2016). The evolutionary history was inferred by using the maximum likelihood method based on the w/freq. model (Jones et al. 1992). The tree with the highest log likelihood (−1301.37) was chosen. Initial tree(s) for the heuristic search were obtained automatically by applying Neighbor-Join and BioNJ algorithms to a matrix of pairwise distances estimated using a JTT model, and then selecting the topology with superior log likelihood value. The analysis involved 30 amino acid sequences. All positions containing gaps and missing data were eliminated. There was a total of 105 positions in the final dataset.

## Results

The Illumina amplicon sequencing resulted in a total of 1275 250 amplicon sequences across all treatments, and after quality filtering, the number was reduced to 1097 148 sequences. The total number of filtered sequence-reads present in each sample were between 17 650 and 43 870, with a mean of 28 870 reads. A total of 614 ASVs were identified. The majority of the sequences belong to the domain bacteria, with only a single ASV represented by 12 reads in one of the Field treatment samples classified as archaea. 25 ASVs, making up 17.3% of the total reads were classified as Eukaryota, which comprised mainly plastids of seaweeds (brown, red, and green) as well as diatoms (24 ASVs) and one mitochondrial ASV classified as Oomycota. A dominant bacterial ASV, ASV 1, accounted for 44% of all sequence reads (54.1% of bacterial reads) and was present in all samples. A BLAST search revealed that ASV no. 1 had 100% sequence identity with cultured strains of the species *Psychromonas marina* (*Gammaproteobacteria*). In addition to ASV 1, a handful of other ASVs were shared among all or most of the samples analyzed, regardless of treatments. These shared, or “core” ASVs are summarized in Table 1. The complete 16S rRNA dataset can be found in the Supplementary online material, including ASV read counts, taxonomic classification of ASVs, and sample metadata (SD1) as well as the DNA sequences of all ASVs in fasta format (SD2).

**Table 1.** Shared ASVs among all or most (>90%) of the samples.

ASV #	Phylum/class	Genus	Average relative abundance (%)	Frequency (% of samples)
ASV 1	*Gammaproteobacteria*	*Psychromonas*	54.1	100
ASV 9	*Gammaproteobacteria*	Unclassified	2.4	100
ASV 4	*Bacteroidetes*	*Lutibacter*	4.1	94.4
ASV 7	*Bacteroidetes*	*Prolixibacter*	2.6	91.7
ASV 8	*Fusobacteria*	*Propionigenium*	2.4	91.7

*Gammaproteobacteria* (to 84% consisting of ASV no 1 *P. marina*) dominated the sea urchin intestinal microbiota in nearly all sampled individuals, yet this dominance was most pronounced in the sea urchins receiving seaweed, regardless of the seaweed species they were fed (Fig. 1A). Starved (Control treatment) sea urchins and those sampled directly after collecting in the field (Field treatment) displayed relatively distinct community composition, well separated from the seaweed-fed treatments (*Fucus, Palmaria*, and *Saccharina*), which were more overlapping in the nMDS ordination (Fig. 1B). Treatment explained 40% of the variation in the community composition, overwhelming the effect of experimental tank (4% of variation explained) according to PERMANOVA (Table 2). A pairwise PERMANOVA analysis indicated that all treatments were significantly different from each other (*P* < .05).

**Table 2.** Results of the multivariate permutational analysis (PERMANOVA) of differences in (Hellinger transformed) bacterial communities between treatments (interaction between the variables treatment and tank are inspected). Treatments: Control, *Fucus, Palmaria*, and *Saccharina*. Formula used: table of ASV ∼ treatment × tank. Significance level: *P* < .001***, *P* < .01**, *P* < .05*, and n.s. = not significant.

	Degrees of freedom	Sums of squares	F. model	*R* ^2^	Pr(>F)	Sign. c.
Treatment	3	2.14	6.31	0.40	0.001	***
Tank	1	0.20	1.76	0.04	0.09	n.s.
Treatment: Tank	3	0.55	1.61	0.10	0.04	*
Residuals	22	2.49		0.46		
Total	29	5.37		1.00		

**Figure 2.**
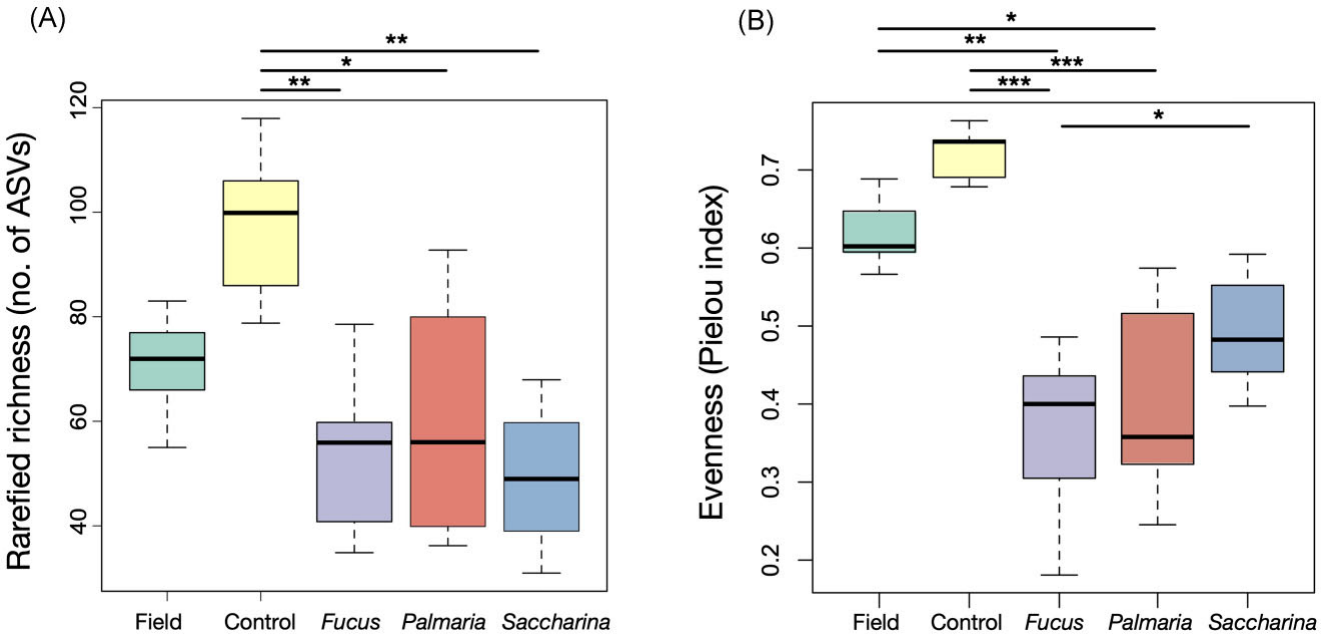
Microbiota diversity was higher in starved (Control) and field-collected sea urchins. The starved sea urchins of the Control treatment displayed the highest (A) rarefied ASV richness and (B) evenness. Box and whisker plots display the median (thick line), quartiles (boxes), and range (whiskers) of the values. Brackets and asterisks above the boxes indicate which of the pairwise comparisons (ANOVA followed by Tukey HSD test) were significant (*P < .05, **P < .001, and ***P < .0001).

Diversity (ASV richness and evenness) was generally lower in the seaweed-fed treatments than in the Control and Field treatments, while there were no significant differences between any of the seaweed-fed treatments. Richness was significantly lower in the seaweed feed (*Saccharina, Fucus*, and *Palmaria*) treatments compared to the Control treatment (*P* < .05), while the Field treatment did not differ significantly (*P* > .05) from the other treatments with regards to richness (Fig. 2A). Evenness was significantly lower in the seaweed feed treatments *Fucus* and *Palmaria* compared to both the Control and the Field treatment (*P* < .05), while these two treatments did not differ from each other, or from the *Saccharina* treatment (Fig. 2B).

**Figure 3.**
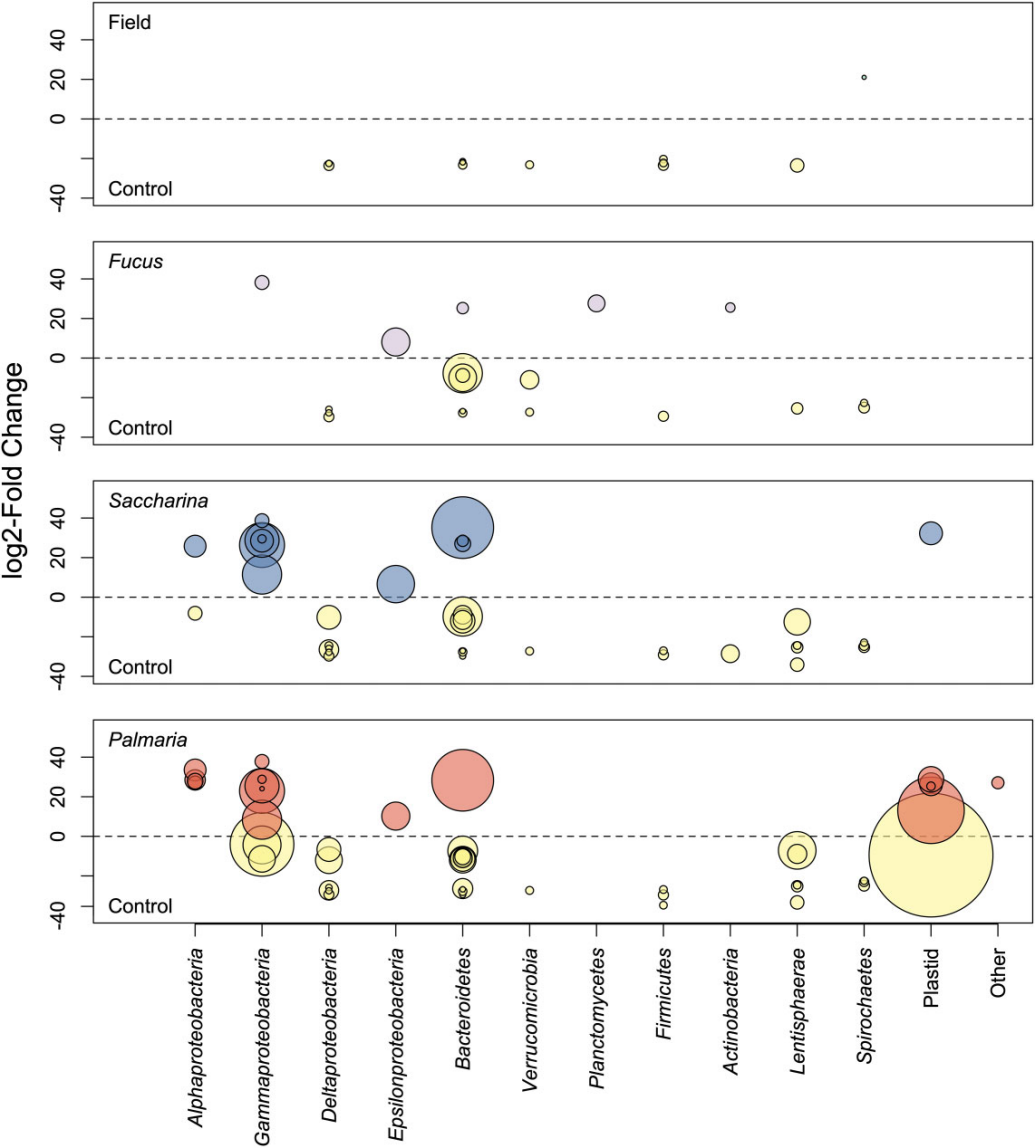
Individual microbial taxa were differentially abundant depending on seaweed diet. ASVs, which were significantly differentially abundant (according to DESeq2 analysis, adjusted *P* < .01) were plotted as bubbles, where bubble radius is proportional to average relative abundance (baseMean) of the ASV across the whole dataset. Positive log2-Fold change indicate higher relative abundance in the respective seaweed diet treatment, while negative values indicate lower relative abundance (ASV more abundant in the Control treatment).

Only one ASV, belonging to the *Spirochaetes*, was more abundant in the Field treatment compared to the Control treatment according to the differential abundance analysis (Fig. 3). The seaweed treatments displayed marked differences to the Control. Notably, ASV 46, related to *Psychromonas* (*Gammaproteobacteria*), was more abundant in all seaweed treatments compared to the Control. Further, ASV 6, related to *Lutibacter* (*Bacteriodetes*), was more abundant in the *Palmaria* and *Saccharina* treatments compared to the Control. Conversely, the Control treatment featured several ASVs related to *Desulfobacterales* (*Deltaproteobacteria*), *Porphyromonadaceae* (*Bacteroidetes*), *Clostridiales* (*Firmicutes*), and *Victivallales* (*Lentisphaerae*), which were less abundant in the seaweed treatments. Several ASVs classified as seaweed plastids were also significantly differentially abundant in the seaweed treatments (Fig. 3). Pairwise comparisons between the different seaweed feed treatments also revealed differentially abundant ASVs (Fig. S1). A complete list of differentially abundant ASVs in each treatment comparison can be found in the online Supplementary material (SD3).

The C:N ratio of the seaweed feed, a proxy for nutritional quality, ranged between 11 and 15.2 (Table S1). *Palmaria* had the highest C:N ratio, followed by *Fucus*, while *Saccharina* had the lowest C:N ratio, and thus the best nutritional quality.

**Figure 4.**
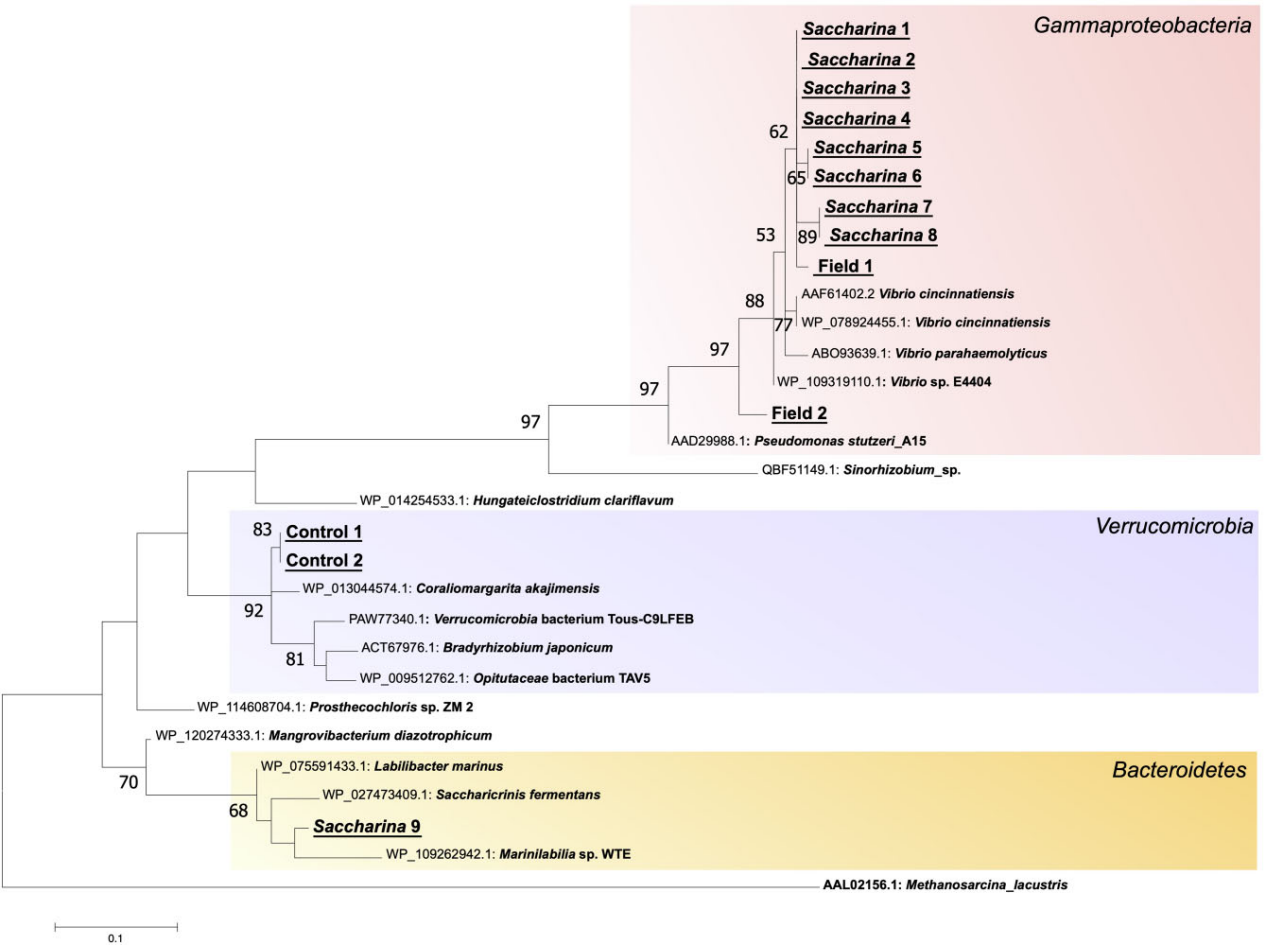
Bacteria capable of N_2_ fixation were present in the sea urchin intestinal microbiome and belonged to diverse phylogenetic groups. The maximum-likelihood gene tree was generated using cloned and sequenced fragments of the *nifH* gene from sea urchins subjected to the control, *Saccharina* (kelp)-fed treatments as well as individuals collected directly from the field. The percentage of trees in which the associated taxa clustered together is shown next to the branches. The tree is drawn to scale, with branch lengths measured in the number of substitutions per site.

Amplification and cloning of the *nifH* gene revealed that bacteria capable of N_2_ fixation were present in the sea urchin fecal pellets and belonged to diverse phylogenetic groups (Fig. 4). Most of the successfully amplified and cloned *nifH* variants came from samples from the *Saccharina* treatment (9 out of 14 clones), and of these eight were related to *Vibrio* spp. (*Gammaproteobacteria*) from previous studies. Two clones from the Field treatment also fell within this clade. Two clones from the starved Control treatment were related to *Verrucomicrobia*, whereas one clone from the *Saccharina* treatment clustered with *nifH* genes from *Bacteroidetes* bacteria. The aligned protein sequences are available in the Supplementary material (SD4).

## Discussion

### Starved and field-captured sea urchins display diverse intestinal microbiota

This study is the first to assess the intestinal bacterial communities of the green sea urchin *S. droebachiensis*, the most important destructive grazer of kelp forests along northern Atlantic coasts (Filbee-Dexter and Scheibling 2014), in response to diet. We sampled fecal pellets, dissected from the large intestine, and amplified the 16S rRNA and *nifH* genes as a representation of the microbiota of the intestines, acknowledging that the microbiota composition changes along the intestinal tract (Hakim et al. 2019), and also contains eukaryotic microbes not addressed here (Holland 2020). We found a bacterial community dominated by a symbiont closely related to *P. marina*, yet also displaying a diverse collection of taxa, especially in sea urchins collected directly from the field and those kept starved in aquaria for several weeks. While we hypothesized that different seaweed as a food source would lead to different intestinal microbiota composition during food degradation (hypothesis 1), we rather found that microbiota composition depended primarily on food availability, regardless of food source. Similar observations were made for larval *S. droebachiensis*, which were reared on different concentrations of microalgal feed in the laboratory. For larvae from the North Sea, which originated from the same location (Drøbak, Norway) as the adult sea urchins investigated in our study, food availability (fed versus starved) had a strong impact on microbial community composition (Carrier et al. 2019).

The green sea urchin microbiota described here shared several features with the previously investigated purple sea urchin intestinal microbiota, such as the presence of *Psychromonadaceae* and variability depending on resource availability (Hakim et al. 2019, Miller et al. 2021). The higher microbial diversity found in starved specimens could indicate the potential for functional plasticity of the microbiome, as (functionally) diverse microbiota prepare the starved sea urchin for digesting a variety of food types it may encounter. In larval stages of the sea urchin *Lytechinus variegatus*, lower food availability was also associated to higher bacterial diversity (OTU richness; Carrier and Reitzel 2019a), indicating that this feature is not unique to adult sea urchins. Field-captured sea urchins also featured relatively diverse microbiota compared to the seaweed-fed treatments, which may reflect the lower food availability typical for the winter conditions they were sampled from, or a more varied diet compared to our laboratory conditions. However, in sea urchin barren grounds in South Korea, the severity of the barren ground, which is related to starvation, did not influence gut bacterial diversity although it did influence microbiota composition (Park et al. 2023). In that study, gut tissue (not fecal pellets) was analyzed, which may have influenced the selection of microbiota captured via their sampling approach. We also collected intestinal tissue samples from our experiment, yet obtained poor PCR amplification results compared to fecal pellet samples (results not shown), which may suggest that intestinal tissue devoid of fecal material harbors relatively low bacterial biomass.

### Algal food intake promotes a dominant symbiont related to *P. marina*

In a feeding experiment, we presented the sea urchins with three different types of seaweed in a no-choice fashion. In all cases, the availability of seaweed led to an increase in abundance of a dominant symbiont, *Psychromonas* ASV 1, which was detected in all samples. *Psychromonas* sp. has also been detected in *S. purpuratus* (Hakim et al. 2019, Miller et al. 2021) and *S. intermedius* (Zhang et al. 2014), and was abundant especially in gut digesta and fecal pellets, analogous to our sample material. Thus, this symbiont appears to be a common feature of the intestinal microbiota of sea urchins belonging to the *Strongylocentrotus* genus, which could point toward important symbiotic functions. In addition to ASV 1, several other *Psychromonas* ASVs were detected in our study. One of them, ASV 46, was consistently more abundant in the seaweed treatments, indicating that it could play a role as a nutritional symbiont. Interestingly, several *Psychromonas* OTUs were also detected in association with eggs of *S. droebachiensis*, but decreased in abundance following fertilization and during larval development, and were suggested to play an important role in early developmental processes (Carrier and Reitzel 2019b). However, in the data of another study on larval *S. droebachiensis* from the North Sea, we did not find *Psychromonas* sp. to be abundant community members when we reanalyzed the data of that study (results not shown; Carrier et al. 2019), indicating that this is a relevant symbiont especially in adult sea urchins. Different strains of *Psychromonas* sp. have also been described as associated with deep sea amphipods, where their reduced genomes indicate a close symbiotic relationship with their hosts (Zhang et al. 2018).

However, *Psychromonas* sp. are not only found in association with sea urchins and other marine invertebrates (Gobet et al. 2018), but are also routinely detected in a variety of marine environments, including on living (Lu et al. 2023, Syukur et al. 2024) and on degrading seaweed (Lozada et al. 2023, Zhang et al. 2024). Therefore, it is possible that this symbiont enters the sea urchin via its diet, and is more or less transient. *Psychromonas marina* was originally isolated from cold water off the coast of Japan and is a facultative anaerobe capable of hydrolyzing alginic acid, a component of brown algal cell walls (Kawasaki 2002). The functional capacity of this symbiont in sea urchins remains enigmatic and could not be addressed further with currently available data, yet it is plausible that it is a nutritional symbiont that may assist the sea urchin in degradation and digestion of seaweed biomass, as such activity has been described for other sea urchin intestinal symbionts (Lasker and Giese 1954, Huang and Giese 1958, de Ridder and Foret 2001). Future investigations should for example uncover genomic or transcriptomic information from the symbiont to elucidate its activity inside the sea urchin intestine or isolate the symbiont in pure culture allowing direct physiological investigation.

### Differentially abundant microorganisms suggest plasticity of intestinal microbiota depending on diet

Differentially abundant ASVs were identified in every treatment compared to the (starved) Control. However, the comparison of the Field and *Fucus* treatments with the Control revealed relatively few differentially abundant ASVs. This may be due to the environment that the sea urchins were collected from which contained abundant *F. serratus*, which likely served as a food source for the Field sea urchins (Marita Helgesen, personal observation). The 10-day aquarium experiment (in addition to 10 days of prestarvation) was apparently not long enough to completely clear the gut contents of the sea urchins in the (starved) Control treatment. Thus, the sampled fecal pellets of the Control sea urchins likely also reflected a diet rich in *Fucus*.

In the remaining seaweed-fed treatments (*Saccharina* and *Palmaria*), a few ASVs stood out, such as ASV 81 (*Rhodobacteriales* and *Alphaproteobacteria*), ASV 46 (*Psychromonas* and *Gammaproteobacteria*), and ASV 6 (*Lutibacter* and *Bacteroidetes*). The latter one was also part of the shared microbiota (present in >94% of samples) and was relatively abundant (>4%). These ASVs, in addition to the omnipresent ASV 1 (*P. marina*) may be good examples of the plasticity of the sea urchin microbiome in response to food availability, which may reflect an important adaptation to a fluctuating marine environment. Several strains of *Lutibacter holmesii* were isolated from the sea urchin *S. intermedius*, and displayed unique repertoires of carbohydrate-degrading enzymes (Nedashkovskaya et al. 2015), which further strengthens the impression that the genus *Lutibacter* contains important sea urchin symbionts.

Microbiome plasticity in sea urchins has been described previously, for example in the context of temperature gradients (Ketchum et al. 2021). Interestingly, in this study, one of the microbial genera identified as plastic in response to temperature in the sea urchin *Echinometra* sp. EZ from the Persian Gulf/Gulf of Oman, *Propionigenium*, was part of the shared microbiota in our study, present in >90% of samples. Plasticity in response to food availability has also been observed in *S. droebachiensis* larvae, yet geographic origin and rearing location had an even stronger influence on microbial community composition in that case (Carrier et al. 2019). This indicates that the surrounding environment and its microbiota makes an important contribution to the plasticity of the sea urchin microbiome, which likely also applies to adult sea urchins. The microbiota of ingested food is no doubt a main influence on the intestinal microbiome of adult sea urchins, and it remains to be investigated to what extent sea urchins depend on a specific set of resident microbial symbionts, or symbiotic functions, in the environment (Hammer et al. 2019).

Unfortunately, we cannot reliably assess the influence of microbiota associated to the seaweed feed in our study, as we did not analyze the seaweed feed separately. It is likely that some of the differentially abundant ASVs represent detection of bacterial cells (or undigested DNA) passing through the sea urchin gut along with the feed. This assumption is supported by the detection of seaweed plastid ASVs, such as the *P. palmata* plastid in sea urchins that were fed this seaweed. Apparently, the feed, and therefore likely the associated bacteria, were incompletely digested during our experiment. One example of an ASV, which was likely originating from the seaweed feed is ASV 125 (*Rhodopirellula* and *Planctomycetes*), which was relatively more abundant in the *Fucus* treatment compared to the Control treatment. *Rhodopirellula* and related planctomycetes are routinely detected and abundant on brown seaweed surfaces, including *Fucus* sp. (Bengtsson and Øvreås 2010, Wiegand et al. 2021). However, it is important to note that seaweed-associated bacteria, which pass through the sea urchin intestines may also play a role in seaweed degradation and thereby interact with the sea urchin host.

### N_2_-fixing microorganisms were detected in both starved and fed sea urchins

We also hypothesized that nitrogen fixation is an important bacterial symbiont trait during seaweed digestion (hypothesis 2) based on the high C:N ratio of the diet and the previous detection of N_2_-fixation in association with *S. droebachiensis* (Guerinot and Patriquin 1981a). We analyzed the *nifH* gene from a selected set of samples of starved, field-captured, and kelp-fed sea urchins (*Saccharina* treatment). This demonstrated several lineages of *nifH*-containing microorganisms within the *Gammaproteobacteria, Verrucomicrobia*, and *Bacteroidetes*. The majority (10 out of 13) of the clones were related to *Vibrio* sp., which agrees well with the earlier isolation of an N_2_-fixing strain belonging to this genus from *S. droebachiensis* (Guerinot and Patriquin 1981b). Unfortunately, we cannot link the presence of a *nifH* gene to the 16S rRNA-based ASVs with our approaches. Therefore, we are unable to conclude whether any of the dominant symbionts possess the capacity of N_2_ fixation, or whether this process played a role in our experiment. Sequencing and assembly of the genomes of dominant symbionts, e.g. based on metagenome data, would have the potential to more accurately link taxonomic identity to functional capacity of sea urchin symbionts.

### Future perspectives

Our feeding experiment represent the first detailed microbiota data for adult *S. droebachiensis* exposed to different diets and therefore provide a baseline for future studies aimed at disentangling the mechanisms of sea urchin seaweed degradation. However, a number of studies have already addressed the gut microbiota of different sea urchins prior to the advent of high-throughput sequencing technologies using e.g. cultivation-based approaches and process-based measurements (summarized in de Ridder and Foret 2001). Whereas these studies were often methodologically limited in their ability to accurately describe the microbial communities inhabiting sea urchins, they provided invaluable data on microbial functional activity through classical methods such as acetylene reduction assays to measure N_2_ fixation (Guerinot and Patriquin 1981a). In addition, key bacterial symbionts were often isolated in culture enabling detailed physiological characterization (Sawabe et al. 1995). There is enormous untapped potential in combining these more classical approaches with contemporary 'omics-based techniques to address the functional role of the sea urchin microbiome in the ecosystem, for example in order to better understand catastrophic sea urchin grazing events.

Kelp, as well as other seaweeds and seagrasses are key producers of blue carbon (Krause-Jensen et al. 2018), which makes their grazers, such as sea urchins, major players in coastal carbon sequestration (Wernberg and Filbee-Dexter 2018). By extension, a dominant symbiont such as *P. marina*, which likely plays an important role in sea urchin food degradation, can also have a disproportional influence on marine carbon cycling. Field experiments are needed to determine the identity and functional potential of symbionts *in situ* during active grazing events. In addition, controlled laboratory experiments involving inoculation of sea urchins with specific symbionts and assessing efficiency of kelp digestion could shed light on host–microbe interactions and organic carbon degradation in the sea urchin microbiome. This study may inform such future investigations, since it highlights important factors, which control bacterial diversity and reveals the identity of a dominant symbiont of the green sea urchin.

## Supplementary Material

fiaf006_Supplemental_Files

## Data Availability

Raw amplicon sequence data has been uploaded to the European Nucleotide Archive under the accession number PRJEB54963. The *nifH* sequences have been submitted to GenBank under accession numbers OP380433–OP380446. Processed data, including ASV tables, taxonomic classification of ASVs, and associated metadata are available as supplementary data. R code used for the analysis of this data can be found at https://github.com/miamynta/sea_urchin.
