# Supplementary material for: Sea urchin intestinal bacterial communities depend on seaweed diet and contain nitrogen-fixing symbionts
Source: FEMS Microbiol Ecol. 2025 Jan 14;101(2):fiaf006. doi: 10.1093/femsec/fiaf006 (PMC11797059; doi:10.1093/femsec/fiaf006)
Supplement: fiaf006_Supplemental_Files [file fiaf006_supplemental_files.zip › FigS1.pdf]

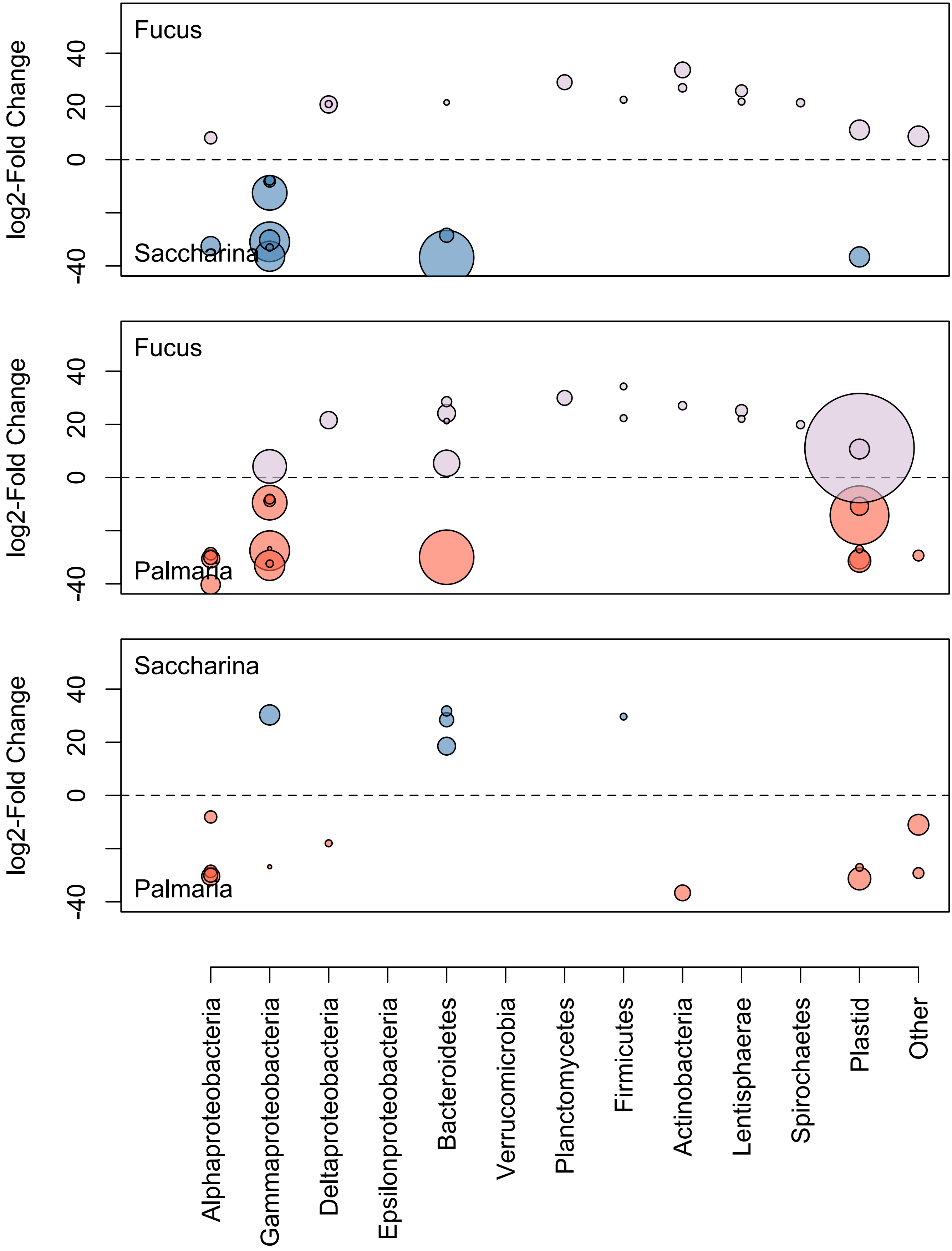

Fucus
Saccharina
Fucus
Palmaria
Saccharina
Palmaria
log2-Fold Change
40
20
0
-40
Alphaproteobacteria
Gammaproteobacteria
Deltaproteobacteria
Epsilonproteobacteria
Bacteroidetes
Verrucomicrobia
Planctomycetes
Firmicutes
Actinobacteria
Lentisphaerae
Spirochaetes
Plastid
Other
