# Supplementary material for: Sea urchin intestinal bacterial communities depend on seaweed diet and contain nitrogen-fixing symbionts
Source: FEMS Microbiol Ecol. 2025 Jan 14;101(2):fiaf006. doi: 10.1093/femsec/fiaf006 (PMC11797059; doi:10.1093/femsec/fiaf006)
Supplement: fiaf006_Supplemental_Files [file fiaf006_supplemental_files.zip › Table_S1.docx]

**Table S1.** Carbon and nitrogen content of the algal feed.

| Alga | Mean N % | Mean C % | C:N ratio | Days of storage |
| --- | --- | --- | --- | --- |
| *Fucus serratus* | 2.45 | 35.72 | 14.60 | 4 |
| *Palmaria palmata* | 2.46 | 37.37 | 15.21 | 9 |
| *Saccharina latissima* | 2.62 | 28.80 | 11.00 | 9 |
